# Clinicopathological characteristics and prognostic factors for intrahepatic cholangiocarcinoma: a population-based study

**DOI:** 10.1038/s41598-021-83149-5

**Published:** 2021-02-17

**Authors:** Tian-hua Yu, Xin Chen, Xuan-he Zhang, Er-chi Zhang, Cai-xia Sun

**Affiliations:** 1 Department of Blood Transfusion, China-Japan Union Hospital of Jilin University, Changchun, Jilin China; 2 Department of Radiology, the First Hospital of Jilin University, Changchun, Jilin China; 3 Shihezi University, Shihezi, Xinjiang China; 4 Rehabilitation Medicine Department, China-Japan Union Hospital of Jilin University, Changchun, Jilin China; 5 Department of Gastrointestinal Colorectal and Anal Surgery, China-Japan Union Hospital of Jilin University, Changchun, Jilin China

**Keywords:** Cancer, Oncology, Risk factors

## Abstract

We aimed to explore the clinicopathological features and survival-related factors for intrahepatic cholangiocarcinoma (ICC). Eligible data were extracted from the Surveillance, Epidemiology and End Results (SEER) database from 2004 to 2015. Totally, 4595 ICC patients were collected with a male to female ratio of nearly 1:1. The higher proportion of ICC patients was elderly, tumor size ≥ 5 cm and advanced AJCC stage. Most patients (79.2%) have no surgery, while low proportion of patients receiving radiotherapy (15.1%). The median survival was 7.0 months (range 0–153 months). The 5-year CSS and OS rates were 8.96% and 7.90%. Multivariate analysis found that elderly age (aged ≥ 65 years old), male, diagnosis at 2008–2011, higher grade, tumor size ≥ 5 cm, and advanced AJCC stage were independent factors for poorer prognosis; while API/AI (American Indian/AK Native, Asian/Pacific Islander) race, married, chemotherapy, surgery and radiotherapy were independent favorable factors in both CSS and OS. Furthermore, stratified analysis found that chemotherapy and radiotherapy improved CSS and OS in patients without surgery. Age, sex, race, years of diagnosis, married status, grade, tumor size, AJCC stage, surgery, chemotherapy and radiotherapy were significantly related to prognosis of ICC. Chemotherapy and radiotherapy could significantly improve survival in patients without surgery.

## Introduction

Intrahepatic cholangiocarcinoma (ICC) is a primary liver malignancy originated from intrahepatic bile duct epithelial cells, whose incidence is second only to hepatocellular carcinoma (HCC)^1^. In comparison with cancer in the upper one third of biliary tract or the two-thirds located in the common hepatic duct bifurcation (Klatskin tumors), ICC is the most uncommon type of cholangiocarcinomas. Although ICC is rare, most patients are diagnosed at advanced and even lethal stage due to the great challenges in detection and therapy^2^.

Although rare, the incidence of ICC has been rising in the past decades^3^, including Japan, Europe, Asia, North America and Australia^4,5^. However, the knowledge of ICC is currently limited, without clear definition of clinicopathological features as well as outcome^6^. Therefore, in order to make clinicians have a better understanding of this rare disease, it is particularly important to deeply explore the clinicopathological features and prognosis of ICC.

The NCI’s Surveillance, Epidemiology and End Results (SEER) database, the most authoritative and largest cancer registry in North America^7^, covers approximately 30% of the total US population by selecting appropriate locations for representing population diversity^8^. As such, SEER is a valuable database to study such rare tumors^9–11^. In our study, ICC patients were retrospectively collected from SEER database to summarize clinical features and survival for patients with ICC to delineate factors influencing prognosis.

## Materials and methods

### Ethics statement

The access of SEER database was signed by the SEER Research Data Agreement (19817-Nov2018), and relevant data were collected according to approved guidelines. All used data were publicly accessible and did not involve any non-human subjects according to the Office for Human Research Protection; therefore, institutional review board approval was exempted.

### Study population

SEER*State v8.3.6 (released at August 8, 2019) was used to select and determine qualified subjects from 18 SEER regions from 2004 to 2015. ICC patients were identified according to ICD-O-3 site codes C22.1 or C22.0 (intrahepatic bile duct and liver) and ICD-O-3 histological codes of 8160/3^12^. Patients were eliminated if: (1) had more than one tumor; (2) only clinically diagnosed or autopsy or death certificate; (3) without certain necessary clinicopathological data (AJCC stage and surgical style); (4) without prognosis information and cause of death; (5) with unknown marital status and race; (6) died within three months after surgery. The rest of subjects were enrolled as the initial cohort of SEER.

### Covariates and endpoint

Patient features were analyzed according to relevant factors: age (˂ 65, ≥ 65); sex (female, male); race (black, white or API/AI); marital status (unmarried, married); insured status (uninsured/unknown, any medicaid/insured); year of diagnosis (2004–2007, 2008–2011, 2012–2015); grade (grade I/II, grade III/IV, unknown); tumor size (˂ 5 cm, ≥ 5 cm, unknown); 6th edition of AJCC stage (stage I, II, III, IV); surgery (no surgery, local tumor excision/segmental resection, lobectomy/hepatectomy), chemotherapy (no/unknown, yes), radiotherapy (no/unknown, yes). To be specific, unmarried population included divorced or separated, single (never married or having a domestic partner) and widowed^13^. Year of diagnosis was equally divided which was referred to a previous study^14^. The stratification of age and tumor size was also based on previous researches^15,16^. API/AI means American Indian/AK Native, Asian/Pacific Islander. In addition, the staging of cancer is based on the 6th edition of AJCC stage system, which adapted to patients in the SEER database with a diagnosis time of 2004–2015.

Overall survival (OS) and cancer-specific survival (CSS) were taken as the study endpoint. OS was defined as the interval from diagnosis to all-cause death, while CSS referred to the interval from diagnosis to ICC-caused death. The cut-off date was set on November 31, 2018 because it was pre-determined until November 2018 (with death data) in accordance with SEER 2018 submission database.

### Statistical analyses

Univariate analysis was estimated by Kaplan–Meier (K-M) method, followed by assessment of the differences of CSS and OS using log-rank test. Parameters with *P* value ≤ 0.2 in univariate analysis were further evaluated in multivariate Cox proportional hazard model^17^. Stratified Cox regression model was conducted, aiming at assessing the prognostic effects of chemotherapy and radiation in different subgroups stratified by surgery style. SPSS software (SPSS Inc., Chicago, USA, version 19.0) was employed for statistical analysis, and GraphPad Prism 5 was utilized for plotting survival curve. A two-sided *P* < 0.05 indicated statistical significance.

## Results

### Patients’ features

**Figure 1:**
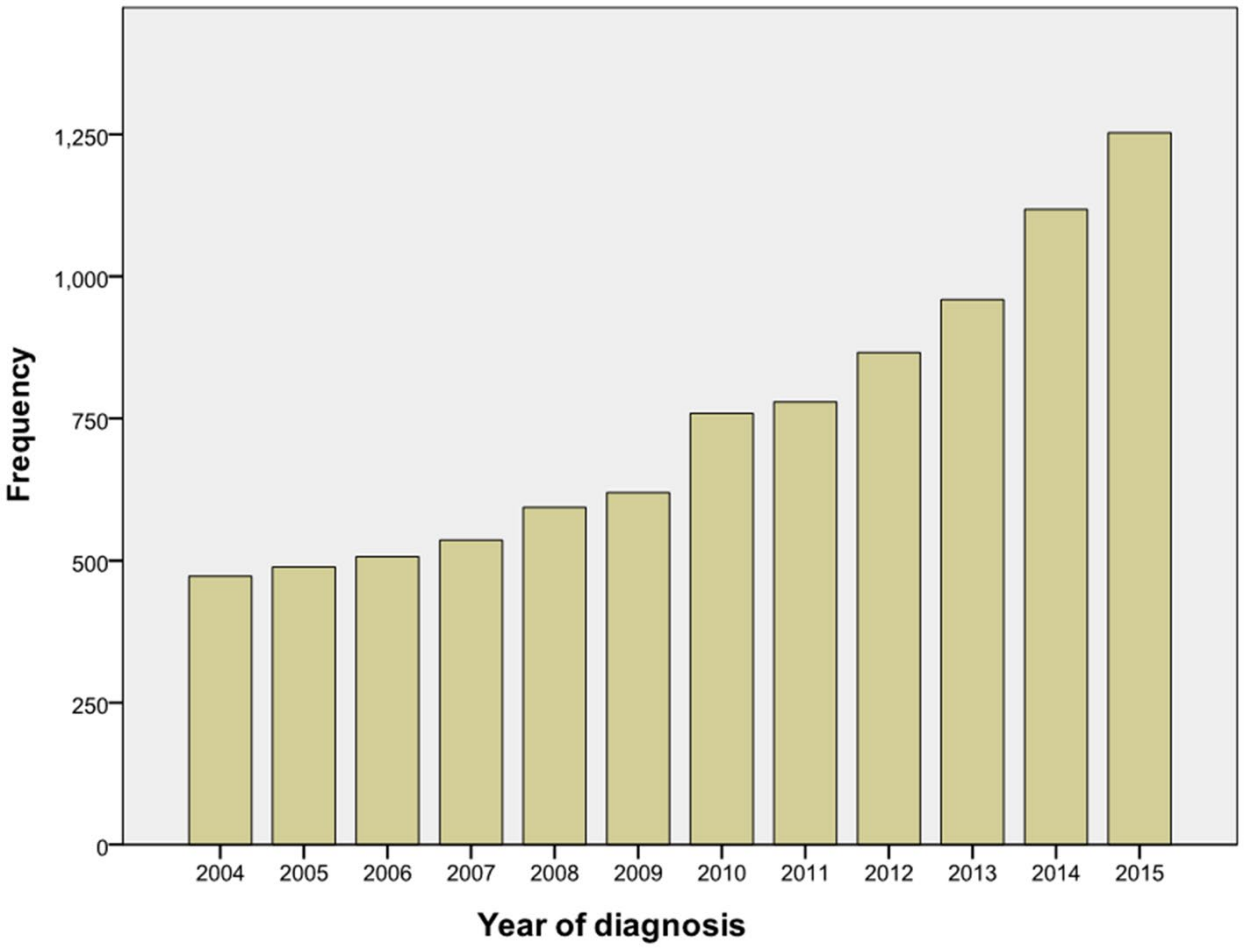
Frequency map of intrahepatic cholangiocarcinoma in SEER database from 2004 to 2015.

**Figure 2:**
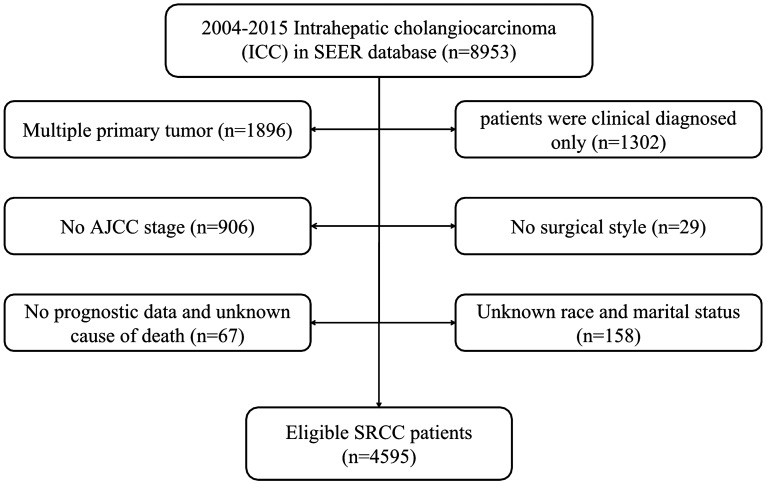
Flow chart for patient selection.

There were 8953 ICC patients from 2004–2015 totally, and the number of patients was increased year by year (Fig. 1). According to the exclusion criteria, 4595 patients were enrolled after screening. The specific screening process was shown in Fig. 2, and features of patients as well as therapeutic regimens were shown in Table 1. The median age was 65 (11–104) years old, with elderly patients (aged ≥ 65 years old) accounting for 51.7% and a male to female ratio of approximately 1:1. Most patients had primary tumors larger than 5 cm (43.4%), and advanced AJCC stage (stage III: 27.0% and stage IV: 48.5%). Most patients lost the surgical opportunity at diagnosis (79.1%); only 20.9% of patients underwent surgical treatment, including radiofrequency ablation and other local treatment. More than half of the patients received chemotherapy (53.7%)while only 15.1% received radiotherapy. Among the patients without surgery, only 2019 (55.5%) and 537 (14.8%) received chemotherapy or radiotherapy respectively.

**Table 1:** The characteristics of the included intrahepatic cholangiocarcinoma patients.

Variables	N (%)
**Age**	
< 65	2219 (48.3%)
≥ 65	2376 (51.7%)
**Sex**	
Female	2261 (49.2%)
Male	2334 (50.8%)
**Race**	
Black	391 (8.5%)
White	3555 (77.4%)
API/AI	649 (14.1%)
**Marital status**	
Unmarried	1805 (39.3%)
Married	2790 (60.7%)
**Insured status**	
Uninsured/unknown	793 (17.3%)
Any medicaid/insured	3802 (82.7%)
**Year at diagnosis**	
2004–2007	918 (20.0%)
2008–2011	1419 (30.9%)
2012–2015	2258 (49.1%)
**Grade**	
Grade I/II	1131 (24.6%)
Grade III/IV	918 (20.0%)
Unknown	2546 (55.4%)
**Tumor size**	
< 5 cm	1035 (22.5%)
≥ 5 cm	1993 (43.4%)
Unknown	1567 (34.1%)
**AJCC stage**	
I	821 (17.9%)
II	307 (6.7%)
III	1239 (27.0%)
IV	2228 (48.5%)
**Surgery**	
No surgery	3637 (79.2%)
Local tumor excision/segmental resection	468 (10.2%)
Lobectomy/hepatectomy	490 (10.7%)
**Chemotherapy**	
No/unknown	2127 (46.3%)
Yes	2468 (53.7%)
**Radiotherapy**	
No/unknown	3899 (84.9%)
Yes	696 (15.1%)

*API/AI* American Indian/AK Native, Asian/Pacific Islander.

### Patient survival and risk factors

The median survival of 4595 patients was 7.0 months (range 0–153 months). The 1-, 3- and 5-year CSS rates were 37.09%, 13.30%, and 8.96%, respectively. Meanwhile, the 1-, 3-and 5-year OS rates were 35.46%, 12.17% and 7.90%, respectively.

Univariate analyses revealed that all variables except race were predictors of CSS (all *P* ˂0.05). Multivariate analysis demonstrated that elderly age (HR 1.175, 95% CI 1.100–1.254, *P* < 0.001), male (HR 1.184, 95% CI 1.110–1.264, *P* < 0.001), diagnosis at 2008–2011 (HR 1.161, 95% CI 1.029–1.310, *P* = 0.015), higher histological grade (HR 1.411, 95% CI 1.277–1.558, *P* < 0.001), tumor size ≥ 5 cm (HR 1.115, 95% CI 1.019–1.219, *P* = 0.018), and advanced AJCC stage (*P* < 0.001) were independent indicators for poor prognosis. Meanwhile, API/AI race (HR 0.846, 95% CI 0.736–0.972, *P* = 0.019), married status (HR 0.904, 95% CI 0.846–0.967, *P* = 0.003), surgery [(local tumor excision/segmental resection) HR 0.322, 95% CI 0.282–0.369, *P* < 0.001 (lobectomy/hepatectomy) HR 0.295, 95% CI 0.268–0.339, *P* < 0.001], chemotherapy (HR 0.425, 95% CI 0.396–0.456, *P* < 0.001) and radiotherapy (HR 0.819, 95% CI 0.748–0.897, *P* < 0.001) were independent favorable indicators. The results of multivariate analysis for OS were similar. The results of univariate factor and multivariate analysis are shown in Table 2.

**Table 2:** Univariate and multivariate analyses of cancer special survival (CSS) and overall survival (OS) for 4595 patients with intrahepatic cholangiocarcinoma.

Variables	CSS			OS		
	Univariate analysis	Multivariate analysis		Univariate analysis	Multivariate analysis	
	*P* value	HR (95% CI)	*P* value	*P* value	HR (95% CI)	*P* value
**Age**	< 0.001		< 0.001	< 0.001		< 0.001
< 65		Reference			Reference	
≥ 65		1.175 (1.100, 1.254)			1.202 (1.127, 1.282)	
**Sex**	< 0.001		< 0.001	< 0.001		< 0.001
Female		Reference			Reference	
Male		1.184 (1.110, 1.264)			1.200 (1.126, 1.279)	
**Race**	0.144		0.048	0.078		0.034
Black		Reference			Reference	
White		0.928 (0.827, 1.041)	0.200		0.913 (0.816, 1.021)	0.110
API/AI		0.846 (0.736, 0.972)	0.019		0.838 (0.732, 0.960)	0.011
**Marital status**	< 0.001		0.003	< 0.001		0.003
Unmarried		Reference			Reference	
Married		0.904 (0.846, 0.967)			0.906 (0.848, 0.968)	
**Insured status**	< 0.001		0.053	< 0.001		0.055
Uninsured/unknown		Reference			Reference	
Any medicaid/insured		0.888 (0.787, 1.002)			0.891 (0.792, 1.003)	
**Year at diagnosis**	0.006		0.001	0.001		< 0.001
2004–2007		Reference			Reference	
2008–2011		1.161 (1.029, 1.310)	0.015		1.162 (1.032, 1.307)	0.013
2012–2015		1.022 (0.908, 1.152)	0.717		1.008 (0.897, 1.133)	0.893
**Grade**	< 0.001		< 0.001	< 0.001		< 0.001
Grade I/II		Reference			Reference	
Grade III/IV		1.411 (1.277, 1.558)	< 0.001		1.397 (1.268, 1.540)	< 0.001
Unknown		1.199 (1.101, 1.305)	< 0.001		1.187 (1.092, 1.290)	< 0.001
**Tumor size**	< 0.001		< 0.001	< 0.001		< 0.001
< 5 cm		Reference			Reference	
≥ 5 cm		1.115 (1.019, 1.219)	0.018		1.086 (0.995, 1.184)	0.065
Unknown		1.252 (1.137, 1.378)	< 0.001		1.227 (1.117, 1.347)	
**AJCC stage**	< 0.001		< 0.001	< 0.001		< 0.001
I		Reference			Reference	
II		1.452 (1.236, 1.706)	< 0.001		1.395 (1.194, 1.631)	< 0.001
III		1.939 (1.740, 2.162)	< 0.001		1.849 (1.665, 2.053)	< 0.001
IV		2.585 (2.322, 2.877)	< 0.001		2.470 (2.227, 2.738)	< 0.001
**Surgery**	< 0.001		< 0.001	< 0.001		< 0.001
No surgery		Reference			Reference	
Local tumor excision/segmental resection		0.322 (0.282, 0.369)	< 0.001		0.330 (0.290, 0.376)	< 0.001
Lobectomy/hepatectomy		0.295 (0.268, 0.339)	< 0.001		0.295 (0.259, 0.337)	< 0.001
**Chemotherapy**	< 0.001		< 0.001	< 0.001		< 0.001
No/unknown		Reference			Reference	
Yes		0.425 (0.396, 0.456)			0.417 (0.389, 0.447)	
**Radiotherapy**	< 0.001		< 0.001	< 0.001		< 0.001
No/unknown		Reference			Reference	
Yes		0.819 (0.748, 0.897)			0.811 (0.741, 0.887)	

*API/AI* American Indian/AK Native, Asian/Pacific Islander, *CSS* cancer‐specific survival, *OS* overall survival.

### Stratified analysis of different surgery style

**Figure 3:**
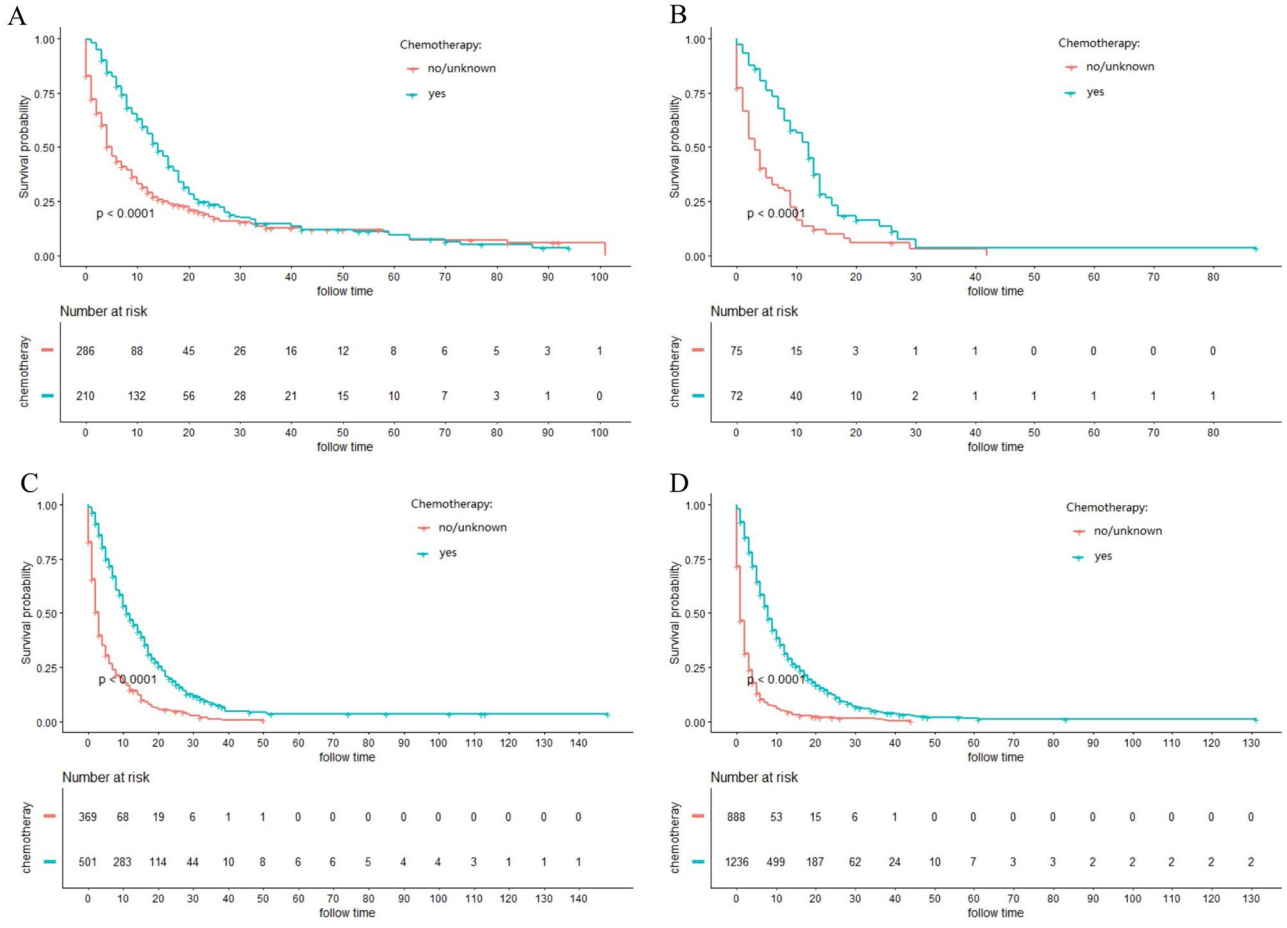
Kaplan–Meier curves for cancer-specific survival (CSS) in different AJCC stage between chemotherapy and no-chemotherapy groups in unresectable ICC patients: (**A**) stage I; (**B**) stage II; (**C**) stage III; (**D**) stage IV.

**Figure 4:**
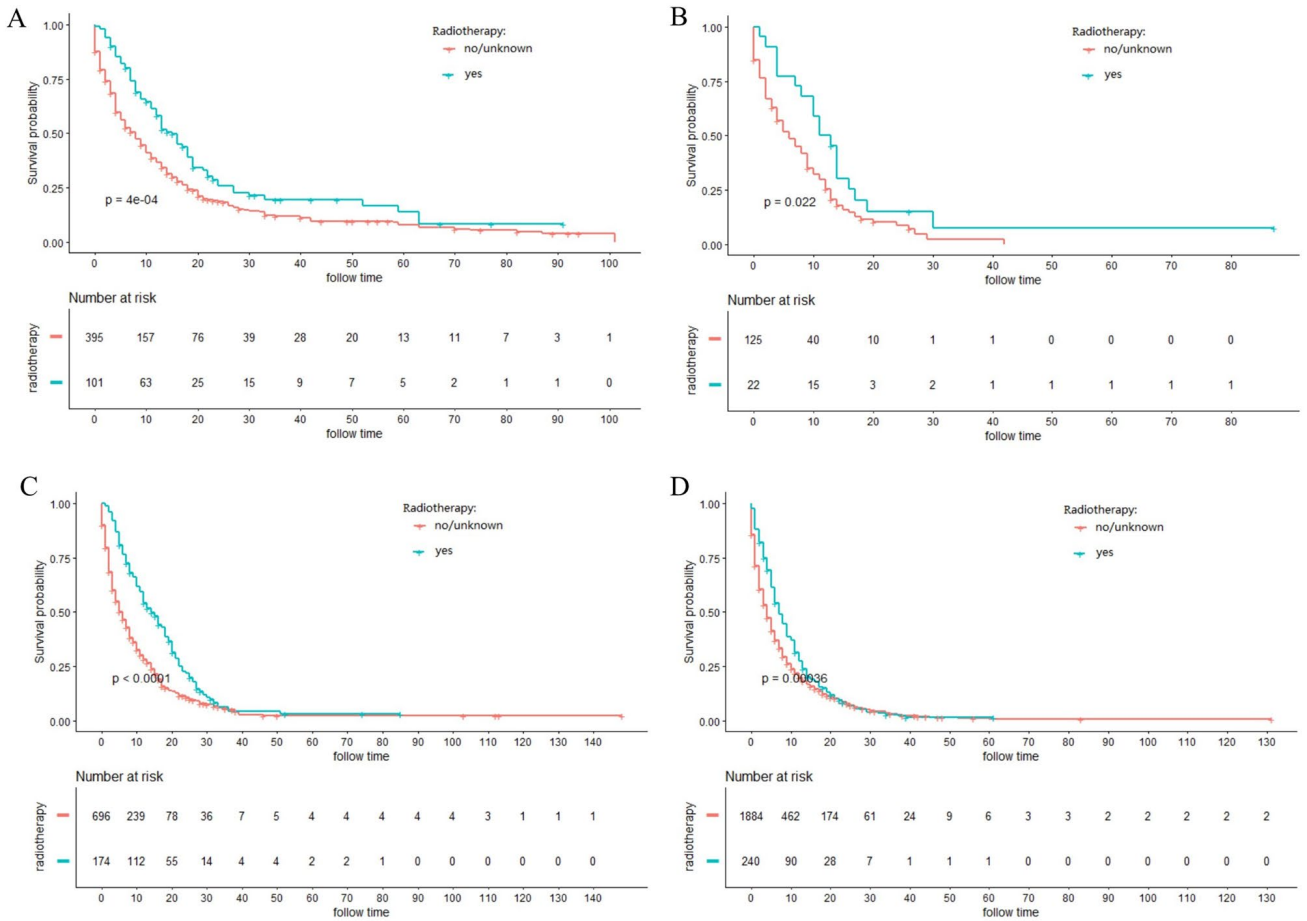
Kaplan–Meier curves for cancer-specific survival (CSS) in different AJCC stage between radiotherapy and no-radiotherapy groups in unresectable ICC patients: (**A**) stage I; (**B**) stage II; (**C**) stage III; (**D**) stage IV.

**Figure 5:**
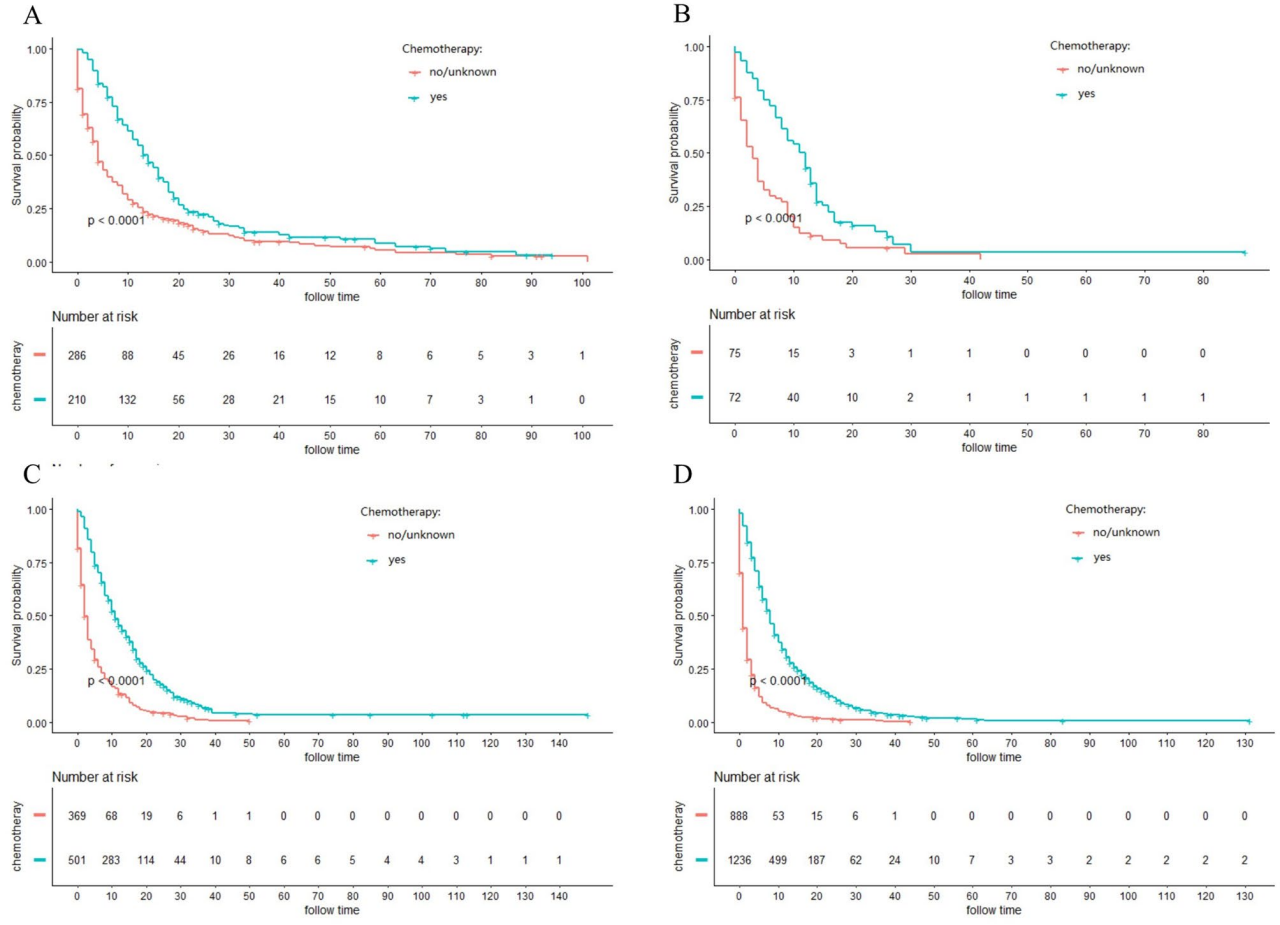
Kaplan–Meier curves for overall survival (OS) in different AJCC stage between chemotherapy and no-chemotherapy groups in unresectable ICC patients: (**A**) stage I; (**B**) stage II; (**C**) stage III; (**D**) stage IV.

**Figure 6:**
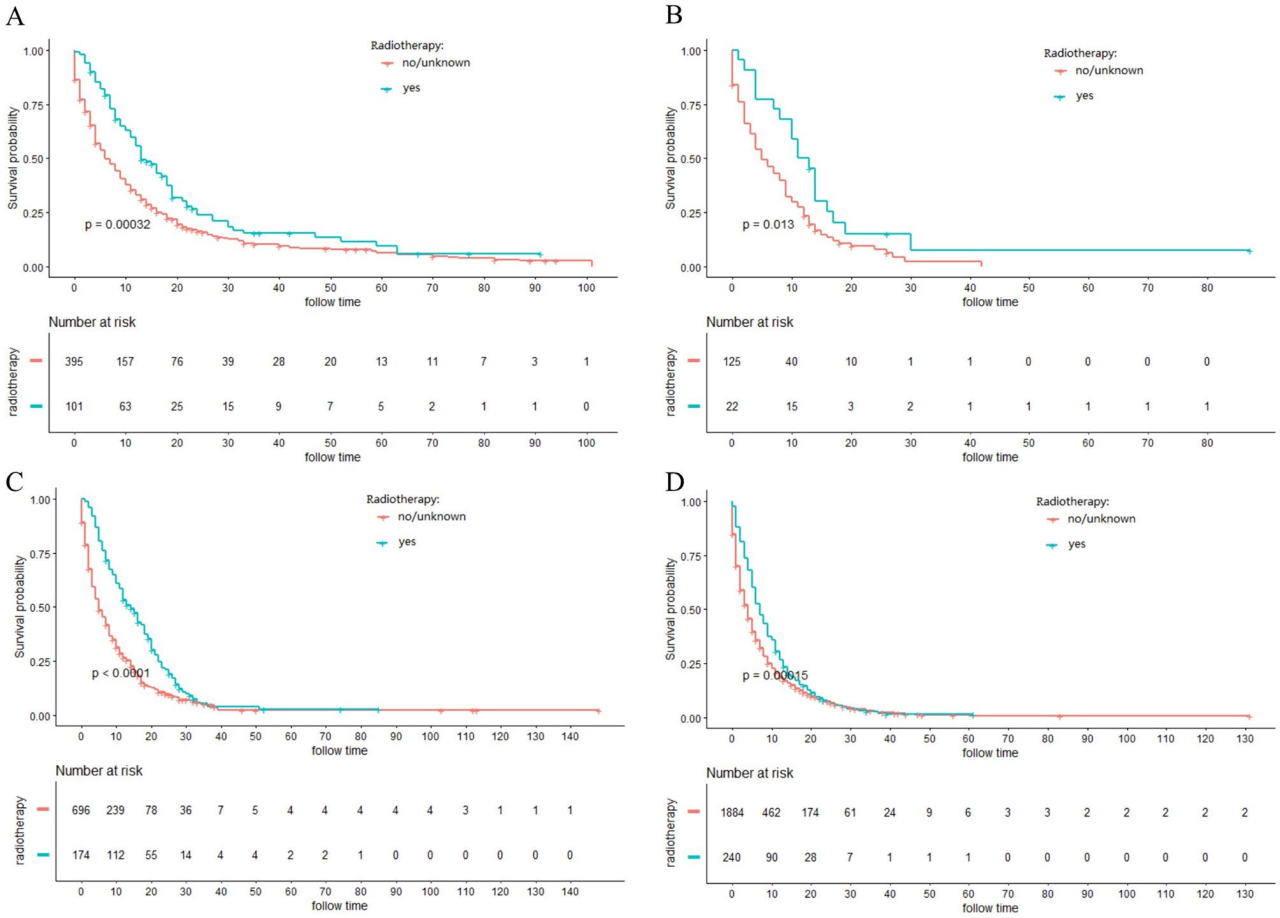
Kaplan–Meier curves for overall survival (OS) in different AJCC stage between radiotherapy and no-radiotherapy groups in unresectable ICC patients: (**A**) stage I; (**B**) stage II; (**C**) stage III; (**D**) stage IV.

The majority of ICC patients (79.1%) were inoperable. In order to investigate the role of chemotherapy and radiotherapy in unresectable ICC patients, these patients were analyzed by K-M curves. Survival curves showed that unresectable ICC patients could significantly obtain survival benefit from chemotherapy or radiotherapy at different AJCC stage in terms of both CSS and OS (all *P* ˂ 0.001) (Figs. 3, 4, 5, 6). For further assessment of prognostic effects of chemotherapy and radiotherapy on patients with different surgery style, stratified Cox regression model was conducted. As demonstrated in Tables 3 and 4, compared to the non-chemotherapy group, chemotherapy group was associated with better CSS and OS in patients who did not receive any cancer-directed surgery (*P* < 0.001). But for patients with surgery did not show significant survival benefit (Table 3). In the stratified analysis of non-radiation group and radiotherapy group, similar results were obtained. Patients in the no surgery group received significant survival benefits after radiotherapy (*P* < 0.001), whether CSS or OS, while patients in the surgery group did not (Table 4).

**Table 3:** Role of chemotherapy related to cancer-specific survival (CSS) and overall survival (OS) in stratified Cox regression analysis.

Surgery style	CSS				OS		
	No chemotherapy	Chemotherapy			No chemotherapy	Chemotherapy	
		HR	*P* value			HR	*P* value
No surgery	Reference	0.76 (0.60, 0.97)	0.026		Reference	0.48 (0.35, 0.66)	< 0.001
Local tumor excision/segmental resection	Reference	1.31 (0.79, 2.16)	0.293		Reference	0.99 (0.59, 1.68)	0.975
Lobectomy/hepatectomy	Reference	0.85 (0.53, 1.35)	0.481		Reference	0.74 (0.46, 1.19)	0.213

Adjustment factors: Age; Sex; Race; Marital status; Insured status; Year at diagnosis; Grade; Tumor size; AJCC stage; radiotherapy.

**Table 4:** Role of radiotherapy related to cancer-specific survival (CSS) and overall survival (OS) in stratified Cox regression analysis.

Surgery style	CSS			OS		
	No radiotherapy	Radiotherapy		No radiotherapy	Radiotherapy	
		HR	*P* value		HR	*P* value
No surgery	Reference	0.76 (0.69, 0.84)	< 0.001	Reference	0.76 (0.69, 0.83)	< 0.001
Local tumor excision/segmental resection	Reference	0.87 (0.61, 1.26)	0.462	Reference	0.82 (0.57, 1.17)	0.276
Lobectomy/hepatectomy	Reference	0.94 (0.68, 1.31)	0.724	Reference	0.91 (0.66, 1.25)	0.558

Adjustment factors: Age; Sex; Race; Marital status; Insured status; Year at diagnosis; Grade; Tumor size; AJCC stage; chemotherapy.

## Discussion

ICC is a subtype of bile duct adenocarcinoma involving liver small ducts^18^, and the second most common primary liver malignancy after HCC^19^. Due to its rarity, few large-scale researches are available for instructive conclusions on proper management for ICC patients^20^. For this purpose, we included a total of 4595 ICC patients to investigate the clinicopathological features and to examine survival-related factors of ICC.

The incidence of ICC has been increased in the US in the last forty years (1973–2012), from 0.44 to 1.18 cases per 100,000^21^, and its incidence is also increasing throughout the world^22^. Previous studies report that ICC patients are elderly, without clear sex differences^23^, which are consistent with our study. Besides, we found that a large proportion of ICC patients had tumor size ≥ 5 cm and advanced AJCC stage. The outcome of ICC is extremely poor, with 5-year OS under 5% from 1975 to 1999^20^. Nevertheless, our study found that the 5-year CSS and OS of ICC from 2004 to 2015 were 8.96% and 7.90%, respectively. From here we see that with the improved modern medical technology, the prognosis of ICC is improving.

Currently, no consensus is achieved on risk stratification for ICC surveillance^24^. Despite hepatolithiasis, viral hepatitis B and C, cirrhosis and primary sclerosing cholangitis reported as risk factors by various researches, data from Eastern and Western countries are not identical^25–27^. Apart from AJCC staging and histological grade, tumor size ≥ 5 cm^24^ and marital status^14^ have also been found to be significant prognostic factors for ICC. Additionally, we found that age, sex and race were also important prognostic factors.

Radical surgery is the only curative treatment, including major liver resection with extended systematic lymph node (LN) dissection^28^, which is recommended by most institutes. However, the resectable rate of ICC is still low, varying from 19 to 74% globally^29^. In our study, only 20.9% of patients underwent surgical treatment. Unresectable ICC patients are generally treated by systemic chemotherapy. ABC-02 trial revealed significant survival advantage in patients with advanced biliary cancer who were treated by gemcitabine/cisplatin combined chemotherapy than those with gemcitabine alone. Other combined regimens included gemcitabine- or fluorouracil-based chemotherapy^6^. NCCN guidelines recommend radiation for subjects with positive regional LN or microscopic tumor margins (R1) following cancer-directed resection^30,31^. And our research found that significant survival benefits of radiation and chemotherapy in non-surgery group according to stratified Cox model (*P* < 0.0001), which were consistent to previous studies^32,33^.

With using advanced technologies like next-generation sequencing (NGS) in ICC, recent research starts to reveal the genetic and molecular processes behind carcinogenesis. The results concluded through empirically studying the genome profiling, epidemiology and experiments offer novel insights into genomic formation, risk factors, cellular origins and constructing tumor microenvironment to the pathogeny of ICC. As a recent retrospective study verifies, the treatment with blockage of Her-2/neu in ICC patients suffering gene amplification has great potential^34^. Immunotherapeutic progress can also offer new opportunities for ICC therapy^35^. After PD-1 inhibitor treatment, a complete response was founded in the chemotherapy refractory metastatic ICC patient who suffers mismatch-repair deficiency (dMMR)^36^. Unfortunately, there is no information on molecular genetic profiles and targeted therapy in the SEER database.

SEER database is the largest publicly accessible and authoritative source on cancer incidence and survival. Therefore, our findings could guide clinical management by using the large-scale, reliable research dataset. As far as we know, our study is largest population-based one to detect prognostic indicators in ICC. Inevitably, there are also several limitations in our study. Firstly, due to the nonrandomized nature of this study, selection bias is inevitable^9,11^. Secondly, certain important factors, including tumor gross type, depth of invasion, status of harvested lymph node, molecular-genetic profiles, metabolic abnormalities of liver and chronic liver disease (viral infection and cirrhosis), were inaccessible in SEER dataset. Thirdly, detailed data on chemotherapy and radiotherapy were not available. Although it is better to obtain more details, we believed that the present available data from SEER database could fit our research objectives very well. Further studies should investigate the above concerns.

## Conclusions

In the present study, we investigated the clinicopathological features and survival of ICC patients. Age, sex, years of diagnosis, grade, tumor size, race, AJCC stage, married status, surgery, chemotherapy and radiotherapy were significantly associated with prognosis. For patients without surgery, chemotherapy and radiotherapy showed significant benefits to improve survival. Hopefully, our findings are of great significance for clinical management and future prospective studies for ICC.
